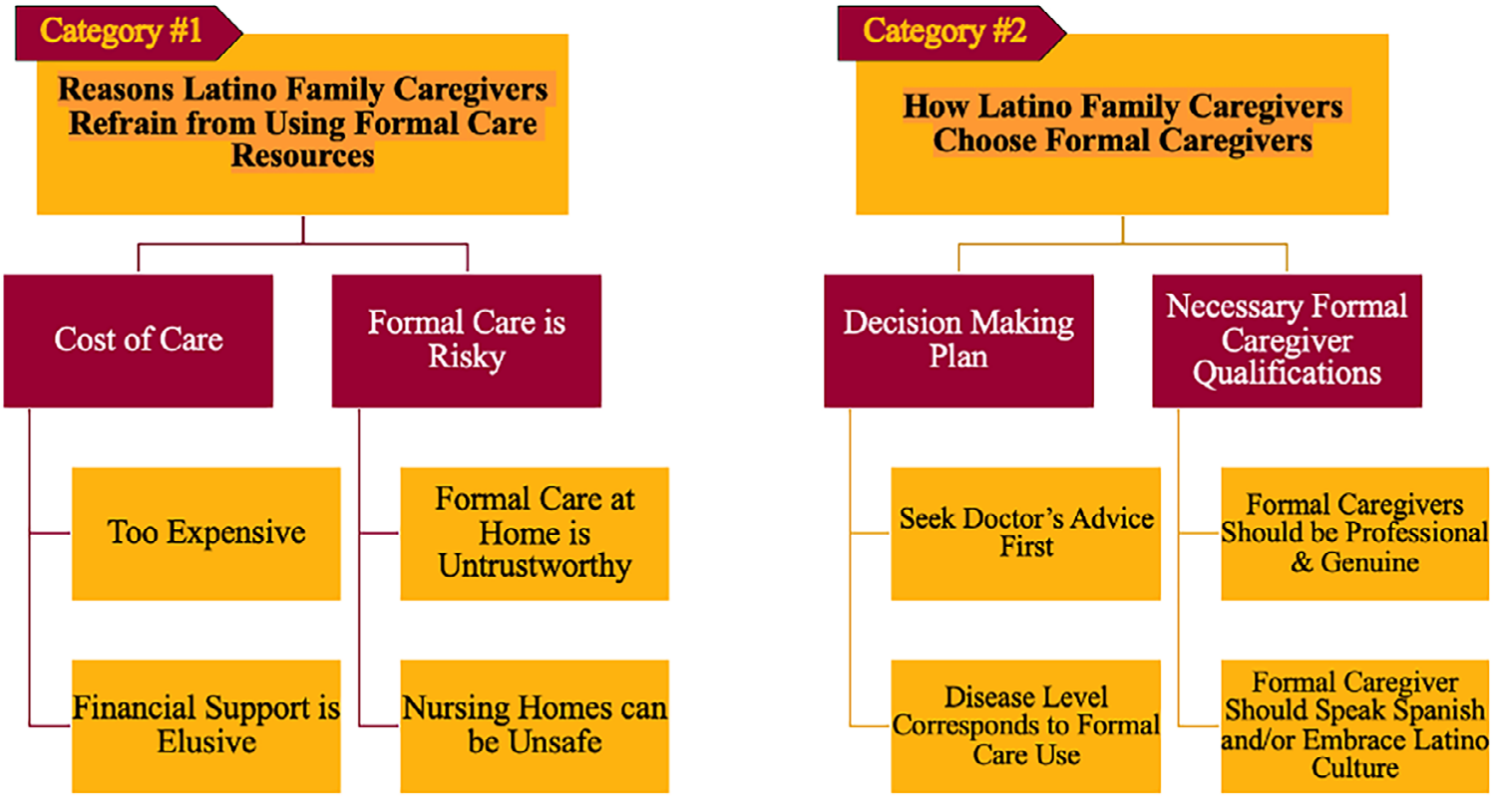# Navigating Cultural Nuances: Understanding and Addressing Formal Care Utilization among Latino Family Caregivers of Older Adults with Dementia

**DOI:** 10.1002/alz.088083

**Published:** 2025-01-09

**Authors:** Alma A Manzo, Maggie Britton, Joahana Segundo, Zachary G Baker

**Affiliations:** ^1^ Arizona State University, Phoenix, AZ USA; ^2^ MD Anderson Cancer Center, Houston, TX USA

## Abstract

**Background:**

In the United States, Latino older adults are 1.5x more likely to develop Alzheimer’s disease than non‐Latino White older adults. Latino Family Caregivers (LFC) maintain home care for longer periods of time but use formal care less. Latino cultural values such as *familismo* influence how LFC provide care, but many LFC report a lack of family assistance that might otherwise compensate for lower formal care usage.

**Methods:**

Phenomenological methodology was employed to understand LFC of people with dementia (*n* = 14). Participants were both female (*n* = 6) and male (*n* = 8) and interviews were conducted in both English (*n* = 8) and Spanish (*n* = 6). These semi‐structured interviews were analyzed via content analysis using NVivo 14 software. A primarily inductive approach was used to code the interviews; however, concepts were influenced by the Ottawa Decision Support Framework.

**Results:**

Two main categories were identified, (1) Reasons LFC might refrain from using formal care and (2) How/when LFC choose formal care. (1) Many LFC conceptualized formal care as untrustworthy (*n* = 10), unsafe (*n* = 8), expensive (*n* = 13), and found financial support elusive (*n* = 12). (2) However, LFC are likely to use at home formal care as disease severity progresses (*n* = 8). Discussing formal care decisions with family was common (*n* = 6), but consulting a doctor for advice was a majority preference (*n* = 11). LFC prized prior experience (*n* = 9) and “genuineness” in formal caregivers (*n* = 5). Lastly, although formal caregivers speaking Spanish was important (*n* = 8), embracing Latino culture was equally valuable (*n* = 8).

**Conclusion:**

Health care professionals (especially doctors) should consider cultural nuances when suggesting formal care to LFC’s. There are preconceived notions that LFC’s do not consider formal care, however if the formal caregiver is trustworthy, professional, and embraces Latino culture, LFC’s are likely to use formal care when the time is right. Future interventions should assist LFC’s in identifying professional and culturally competent formal caregivers and locating financial resources.